# The electromyographic single twitch stimulation for monitoring the effect of rocuronium on vocal cord opening – a randomised controlled trial

**DOI:** 10.1186/s12871-025-03201-z

**Published:** 2025-07-03

**Authors:** Jennifer Herzog-Niescery, Maximilian von der Gönna, Sarah Joline Werner, Thomas Peter Weber, Adrian Iustin Georgevici

**Affiliations:** 1 Department of Anaesthesiology and Intensive Care Medicine, Katholisches Klinikum Bochum, St. Josef Hospital, Bochum, Germany; 2 Ruhr-University Bochum, Department of Anaesthesiology, St. Josef Hospital Bochum, Gudrunstrasse 56, Bochum, 44791 Germany

**Keywords:** Vocal cords, Rocuronium, Single twitch stimulation, Quantitative neuromuscular blocking drug monitoring

## Abstract

**Background:**

The European Society of Anaesthesiology and Intensive Care recommends the use of neuromuscular blocking drugs for tracheal intubation, but the monitoring is difficult, because the parameters are mostly relative values (e.g. Train-of-four ratio), which show no or only weak correlations to the vocal cord aperture. We investigated the predictive effect of the quantitative single twitch (0.3 ms duration supramaximal stimulus, frequency 0.1 Hz) to estimate vocal cord aperture (primary endpoint). Secondarily, we focused on rocuronium dose-related differences between single twitch amplitude and maximum vocal cord aperture.

**Methods:**

Thirty-six adult patients undergoing elective surgery with tracheal intubation using rocuronium were included. Patients received remifentanil and propofol for induction of anaesthesia before the neuromuscular block baseline was measured electromyographically using the single twitch stimulation pattern of the ulnar nerve from the abductor digiti minimi muscle. A video-laryngoscope was inserted to document baseline conditions before the patient received either 0.3 or 0.9 mg/kg IBW rocuronium. The vocal cord area was continuously videorecorded for four minutes, before the trachea was intubated.

**Results:**

Thirty-five patients completed the study; 18 received 0.3 and 17 received 0.9 mg/kg IBW rocuronium. Data showed a strong correlation between single twitch amplitude and vocal cord aperture (bootstrapped Pearson’s coefficients, median ± IQR: -0.58 ± 0.22 in rocuronium 0.3 and -0.74 ± 0.18 in rocuronium 0.9 mg/kg IBW; *p* < 0.001), meaning that the single twitch amplitude may be a reliable predictor of vocal cord opening. The higher rocuronium dose caused a stronger correlation, lower inter-patient variability, and a steeper single twitch decrease, but the effect on the maximum vocal cord aperture was comparable to that in the 0.3 mg/kg IBW rocuronium group.

**Conclusions:**

The quantitative, electromyographic single twitch stimulation pattern can dose-independent predict vocal cord opening after rocuronium administration.

**Trial registration:**

The study was registered at the German Clinical Trials Register on the 10th of July 2020 (DRKS00021433) prior to enrolment of the patients.

## Background

The current guideline from the European Society of Anaesthesiology and Intensive Care recommends the use of neuromuscular blocking drugs (NMBDs) to facilitate mask ventilation and tracheal intubation, which includes, besides open vocal cords, sufficient relaxation of the masseter muscle, pharyngeal and laryngeal muscles, and the diaphragm [1]. It is known that all these muscles have different sensitivity to NMBDs, which is, among others, caused by the composition, contraction time, and diameter of the muscle fibres, the acetylcholine receptor subunit expression, and the local blood circulation [2, 3]. In addition, the overall response to NMBDs depends on the individual patient with a huge interpatient variability, which may be influenced by the cardiovascular circulation time, neuromuscular diseases, hepatic, biliary, or renal insufficiency, electrolyte imbalances, body weight, body temperature, and drug interactions [4]. Thus, ‘readiness for tracheal intubation’ cannot be determined by subjective ‘experience’ or simply be assumed because the anaesthesiologist had waited for a defined time interval after the NMBD was given [1]. This applies in particular if comparatively low NMBD doses are used, e.g. for short surgical procedures.

Regardless of the type of NMBD monitoring (e.g. mechanical or electrical), the train-of-four (TOF) is a well-established stimulation pattern to determine the depth of the neuromuscular block. Here, four stimuli with a frequency of 2 Hz are given. Non-depolarizing NMBDs such as rocuronium cause neuromuscular fade, meaning that the fourth twitch (T4) is weaker than the first twitch (T1). Once a 75% depression of T1 is reached, T4 disappears, while T2 disappears after a 90% T1 depression. As this happens regularly, the depth of the neuromuscular block is typically assessed by the TOF count, which is the number of twitches following TOF stimulation [5]. Patients with a deep neuromuscular block may show no response to TOF stimulation (TOF count = 0); here the post-tetanic count (PTC) with a 5 s stimulus (50 Hz) followed by up to 20 single twitches (1 Hz) is helpful for monitoring deep block. However, the PTC should not be repeated for at least two minutes [5].

Studies tend to state that a TOF count of 0 resulting from N. ulnaris stimulation (response from M. adductor pollicis or M. digiti minimi) corresponds to improved mask ventilation and good intubation conditions [6–8]. However, the TOF pattern is not suitable to judge the exact depth of the neuromuscular block, and the PTC stimulation is not useful in clinical practice due to its long recovery time that does not allow frequent monitoring. Another stimulation pattern is the electromyographic single twitch (ST), which demonstrates an evoked response to an 0.3 ms duration individual supramaximal stimulus at a frequency of 0.1 Hz and that can be repeated every 10 s [5, 9]. The ST amplitude, which is the maximum-minimum after ST stimulation [mV], might be an alternative to monitor a neuromuscular block combined with a shorter measuring interval than the TOF, and could therefore help to identify the ideal intubation time.

In this study we investigated the correlation between ST amplitude (response amplitude) and vocal cord opening (primary endpoint). Secondarily, we intended to compare possible dose-related differences in the ‘ST response amplitude’ and the ‘vocal cord aperture’ after administration of either 0.3 or 0.9 mg/kg ideal body weight (IBW) rocuronium.

## Methods

This randomized, controlled single-centre pilot study was approved by the Scientific Ethics Committee on the 7th of July 2020 (Ruhr-Universität of Bochum, chairperson: Prof. Trampisch, protocol number: 20–6923) and registered at the German Clinical Trials Register on the 10th of July 2020 (DRKS00021433) prior to enrolment of the patients.

This study was conducted between April and October 2023 at a German University Hospital and performed according to the most recent version of the Declaration of Helsinki. Written informed consent was obtained from all patients at least one day before the surgical intervention. This study adheres to CONSORT guidelines.

### Inclusion and exclusion criteria

Patients aged ≥ 18 years with an American Society of Anesthesiologists (ASA) physical status (PS) class I to III scheduled for elective surgery with planned tracheal intubation using rocuronium from the Department of General Surgery were enrolled in this study.

Exclusion criteria included need for rapid sequence induction, evidence for a difficult airway management, any neuromuscular disease, allergy to rocuronium, contractures of the hands, pregnancy, and limited ability to provide study consent.

Stop criteria were a baseline electromyographic response < 5 mV, inadequate face mask ventilation prior to rocuronium administration, an incomplete view on the vocal cord area via video laryngoscopy, and an oxygen saturation < 94% during video documentation.

### Randomization

Patients were randomly either allocated to group rocuronium 0.3 mg/kg IBW or rocuronium 0.9 mg/kg IBW in a 1:1 ratio. The randomization process utilized a computer-generated sequence to ensure equal allocation and minimize bias. The rocuronium syringe was prepared by the study investigator as follows: The rocuronium dose for each patient was calculated according to the patient’s IBW (female: [height in cm minus 100] minus 15%; male: [height in cm minus 100] minus 10%). The syringe was then filled to the 10 ml mark with sodium chloride. The anaesthesiologist was blinded to the study group.

### Study design and setting

Each patient was monitored with electrocardiography, non-invasive blood pressure, pulse oximetry, and an electroencephalogram (Narcotrend®-Compact M, Narcotrend, Hannover, Germany). A TetraGraph® electrode (TetraSens, Senzime AB, Uppsala, Sweden) was placed on the left volar forearm along the ulnar nerve and on the abductor digiti minimi muscle, and connected to the electromyographic neuromuscular monitor TetraGraph® (software version 34 g.22 h.12e; Senzime AB).

**Fig. 1:**
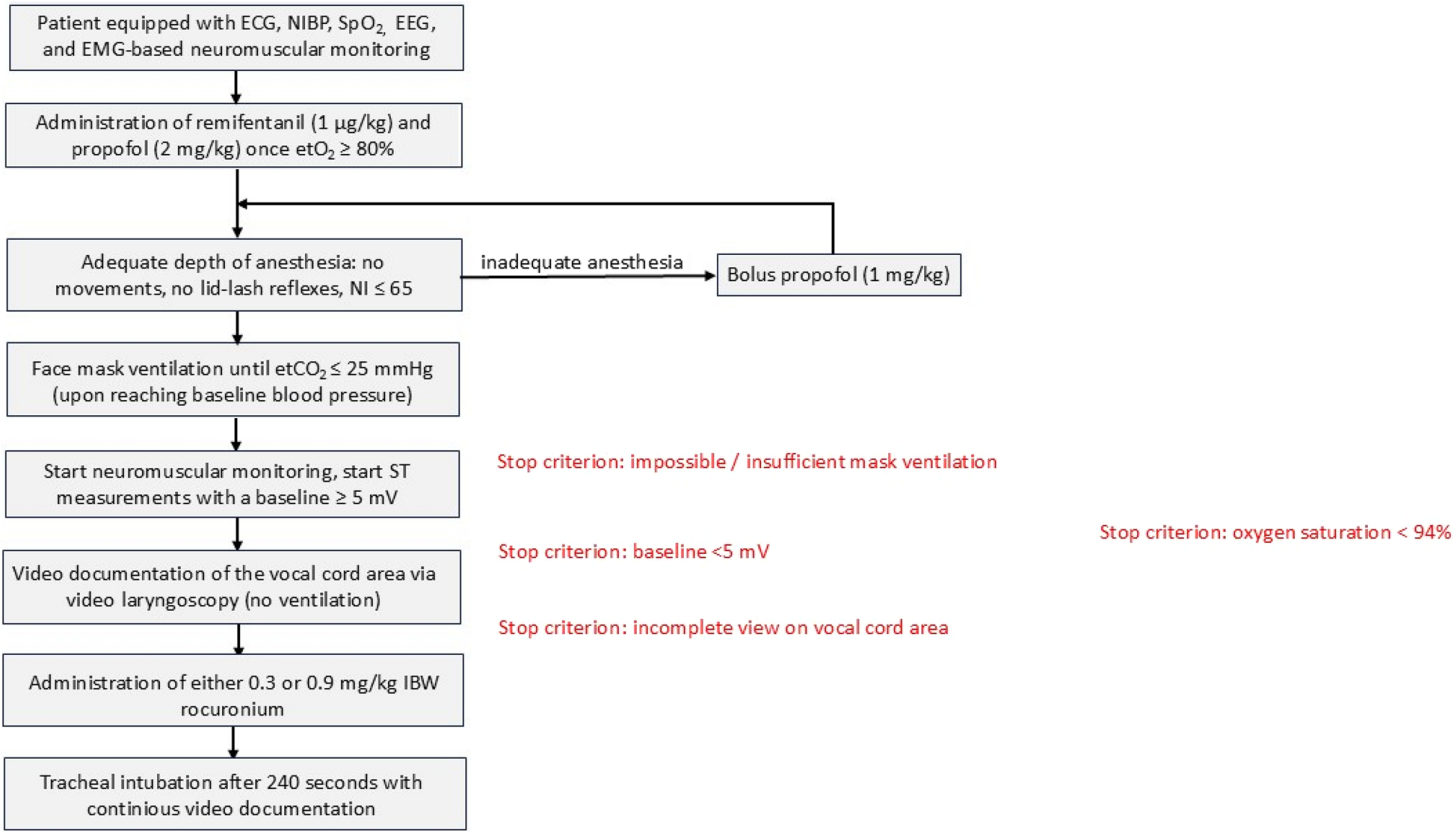
Experimental setup. Once the video laryngoscope was inserted to document the vocal cord starting conditions, either 0.3 or 0.9 mg/kg IBW rocuronium was given. The video laryngoscope remained in situ for 240 s, while the single twitch stimulation pattern was performed every 10 s. Thereafter, the trachea was intubated. An insufficient ventilation via face mask, an EMG neuromuscular monitoring baseline < 5 mV, an incomplete view on the vocal cord area via video laryngoscopy, and a drop of the oxygen saturation < 94% during the observation period were stop criteria. ECG: electrocardiography; NIBP: noninvasive blood pressure; SpO2: saturation of peripheral oxygen; EEG: electroencephalography; etO2: end-tidal oxygen; etCO2: end-tidal carbon dioxide; NI: Narcotrend index; ST: single twitch; mV: millivolts; IBW: ideal body weight

A Drager Primus anaesthesia machine was used to oxygenate the patient via facemask to an 80% end-tidal oxygen saturation. Anaesthesia was then induced intravenously with remifentanil (1 μg/kg) and propofol (2 mg/kg). A Narcotrend index ≥ 65, any patient movements, or a preserved lid lash-reflex were considered as inadequate depth of anaesthesia, and an additional propofol bolus (1 mg/kg) was given. Once anaesthesia was adequate, the patient’s lungs were manually ventilated via face mask until an end-tidal CO_2_of 25 mmHg was reached. The neuromuscular monitor was then started. The baseline was determined (minimum of 5 mV was needed according to the manufacturer recommendations), and the stimulation pattern switched from TOF to ST for at least 3 measurements to establish a stable baseline ST prior to NMBD administration (no inverse fade effect expected in EMG-based devices) [10]. Then, a video laryngoscope (C-MAC®, Storz, Tuttlingen, Germany) was used to document the vocal cord area starting conditions (degree of opening) before the patient received either 0.3 or 0.9 mg/kg IBW. The video laryngoscope was left in situ for 240 s and continuously videorecorded the vocal cord area. Patients were not ventilated. During this time, the ST stimulation pattern at 0.1 Hz was recorded on the interfaced TetraGraph®. After 4 min the trachea was intubated (Fig. 1). The vocal cord area video data were later analysed with the software MATLAB® (MathWorks, Bochum, Germany). The computing of the vocal cord area was performed after tuned auto-segmentation, namely automatically counting the number of pixels in the darker tracheal area. This raw area was then corrected for distance and angle in reference to the fixed arytenoid cartilages.

### Sample size estimation

The required sample size for the primary endpoint was calculated by an a priori power analysis. Considering that the administration of rocuronium is not followed by fasciculations, we expected a moderate correlation (R^2^ > 0.3) between the predictor ‘ST response amplitude’ and the outcome ‘vocal cord opening.’ At a significance level of α = 0.05 and a power 1 − β = 0.8, the one-tailed Pearson’s test required 16 patients in each group. By accounting for 10% potential dropouts, we aimed to include 18 patients per study arm, resulting in n = 36 patients.

### Statistical analysis

To consider patient-specific and inter-individual factors such as skin impedance (electrode), muscle response, laryngeal anatomy, camara angle and its distance to the vocal cords, the vocal cord area and the response amplitude were scaled linearly for each patient between the minimum 0 and the maximum 1. The scaling for vocal cord aperture was computed as follows: (actual area—min(areas))/(max(areas)-min(areas)). As such, the smallest vocal cord aperture becomes zero, whereas the maximum vocal cord aperture is 1 in each patient. The scaling allows the estimation of the correlation coefficients across the variables in the studied cohorts, regardless of their inter-individual differences.

Demographic data, including Body Mass Index, weight, age, and ASA PS classification were collected. Continuous variables following a normal distribution according to Shapiro–Wilk’s test were summarized as mean ± standard deviation (SD), else as median ± interquartile range (IQR). Categorical variables were expressed as percentages. For the analysis of the demography across randomized groups, we used independent samples t-test for normally distributed variables or Wilcoxon rank-sum test for non-normally distributed continuous variables. For categorical variables, Fisher’s Exact Test was applied to 2 × 2 contingency tables, else the Chi-square test.

Our data is characterized by timeseries of paired values collected at 10-s intervals, therefore we expected that data points more closely in time are more probable to be related than those further apart (i.e. autocorrelation), whereas conventional linear models or Pearson’s test require no autocorrelation. Therefore, we statistically investigated the timeseries, without the autocorrelation bias, using either bootstrapped Pearson’s correlation or linear multilevel models (LMM) for the primary and secondary endpoint, respectively.

The primary hypothesis was explored using 1000 bootstrapped Pearson’s correlation coefficients, in each iteration only one pair of variables per patient was stochastically sampled, thus respecting the assumption of data independence. Moreover, bootstrapping is known to provide a more robust estimation of the correlations. We compared the distributions of the coefficients across the two arms using the t-test.

The secondary endpoints were analysed using LMM that account for both inter-individual variance and repeated measurements with inherent autocorrelation. They were used to test if the rocuronium dose is a significant predictor for either the vocal cord opening or the ST response amplitude fading over time, while controlling for random effects due to intra-subject variability. Therefore, two LMM were constructed for each outcome variable: vocal cord area and ST response amplitude. For both outcomes, the initial model included ‘elapsed time’ as a fixed effect and ‘patient’ as a random effect to account for the repeated measures design. This model structure captured the baseline effect of time on the outcomes without considering group differences.

To evaluate the impact of rocuronium administration and its interaction with time, a second model for each outcome incorporated both the main effects of group and elapsed time, as well as their interaction term. The inclusion of the interaction term allowed us to assess whether the rate of change over time differed between groups. Model comparisons were conducted using analysis of variance (ANOVA) and explored the significance of adding the rocuronium dosage and its interaction with elapsed time to the model’s prediction. Furthermore, we visually investigated potential non-linearities in the data with generalized additive regressions.

The analysis was conducted using R version 4.3.1 (R Foundation, Vienna, Austria) [11].

## Results

Thirty-six patients were included in this study, 18 in each group. One patient of group 0.9 mg/kg IBW rocuronium was later excluded due to incomplete data transmission. Their demographic data are presented in Table 1.

**Table 1:** Demographic data. The not normally distributed demographic data, presented as median ± IQR for rocuronium 0.3 versus 0.9 mg/kg IBW, demonstrated no significant differences, and thus suggested comparability between both groups. ASA PS: American Society of Anesthesiologists physical status

	0.3 mg/kg IBW rocuronium	0.9 mg/kg IBW rocuronium	*p*-value
Weight [kg]	79 ± 34	82 ± 17	0.516
Body Mass Index	26.2 ± 7.6	26 ± 3.7	0.705
Age [yr]	56.1 ± 29.7	61.5 ± 22.6	0.494
ASA PS [I/II/III]	6/8/4	2/9/6	0.133

**Fig. 2:**
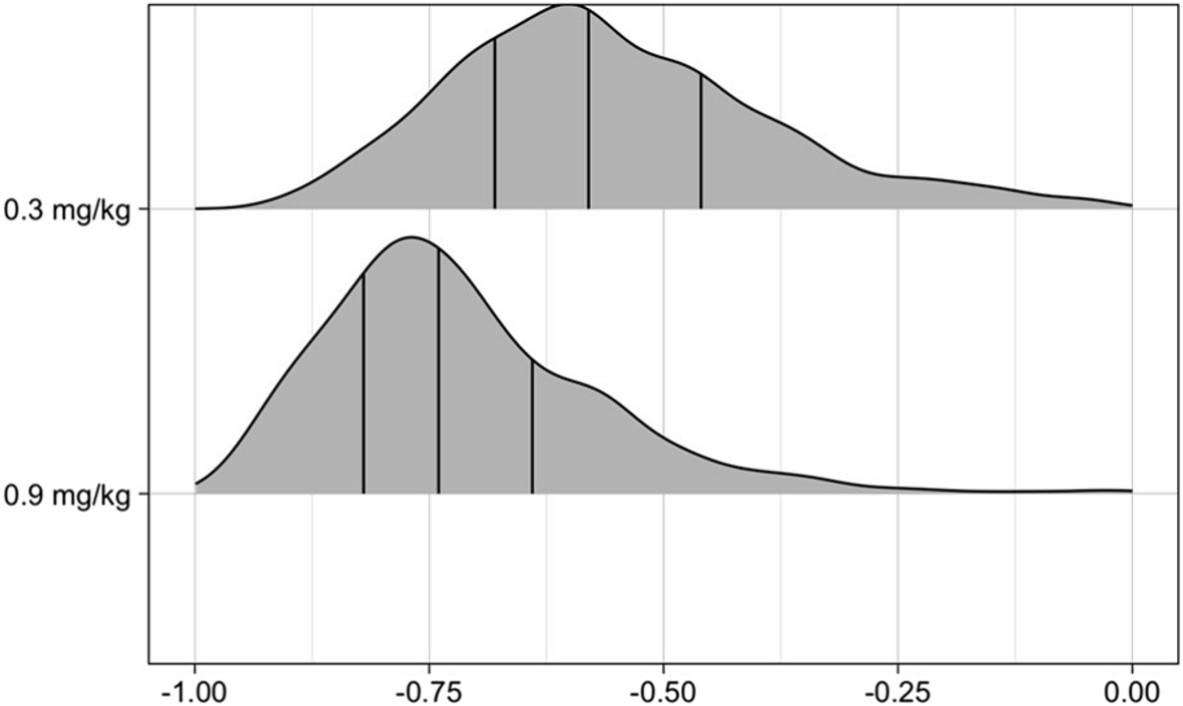
Pearson’s coefficients between ST response amplitude and vocal cord opening per study group. The figure presents the density plot after 1000 bootstrapping iterations for patients receiving either 0.3 or 0.9 mg/kg IBW rocuronium. The vertical lines correspond (from left to right) the 1 st, 2nd, and 3rd quartile. The two distributions differ significantly, the higher rocuronium dose leads to a stronger negative correlation compared to the 0.3 mg/kg IBW group

The analysis of the primary endpoint revealed significant correlation coefficients between the decreasing ST response amplitude and the increasing vocal cord opening. The median ± IQR of the bootstrapped Pearson’s coefficients were −0.58 ± 0.22 in the rocuronium 0.3 mg/kg IBW group and −0.74 ± 0.18 in the rocuronium 0.9 mg/kg IBW group, respectively. The sample size for the primary endpoint was computed for R^2^ > 0.3, corresponding to a median Pearson’s correlation coefficient more negative than –0.54, therefore the bootstrap analysis confirmed our main hypothesis in both groups (Fig. 2).

**Fig. 3:**
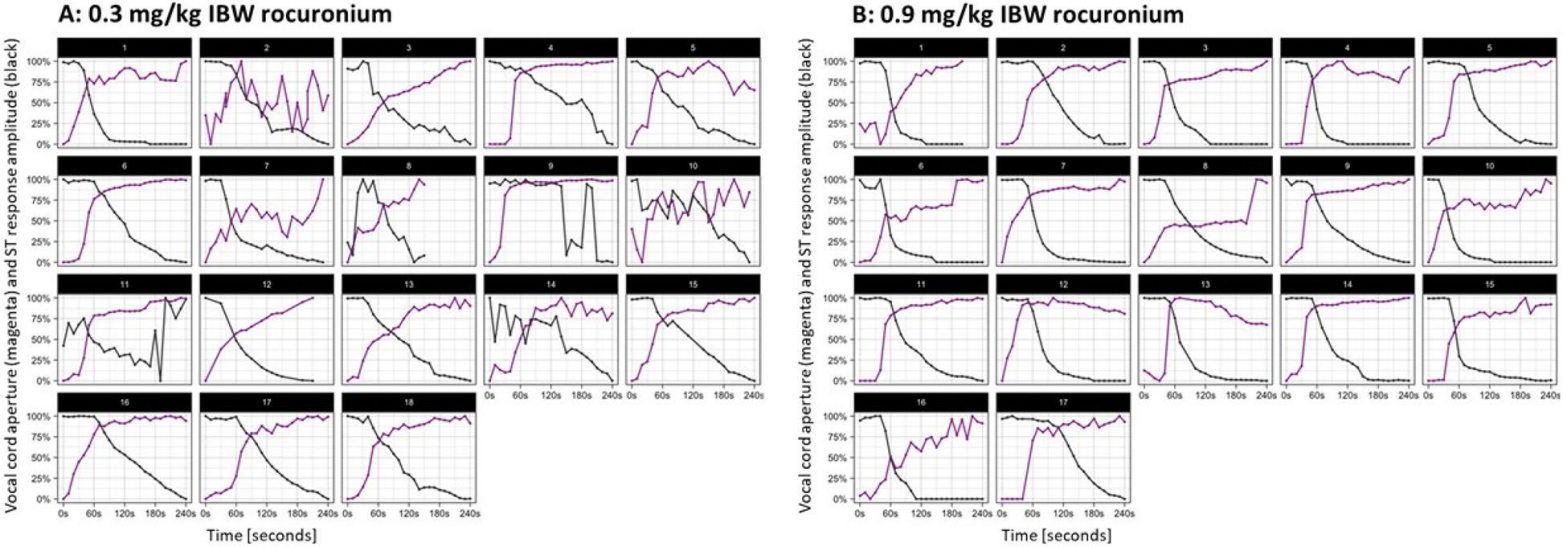
Dose–response dynamic per patient. The vertical axis shows the two scaled (normalized to 0–100% range) parameters ‘vocal cord aperture’ (magenta) and ‘ST response amplitude’ (black). The horizontal axis pictures the elapsed time since the administration of rocuronium (A: 0.3 mg/kg IBW; B: 0.9 mg/kg IBW). The negative/inverse correlation between response amplitude and vocal cord aperture can even visually identified easily

In addition, the t-test demonstrated a significant difference between the two groups (*p* < 0.0001); the correlation between ST response amplitude and vocal cord opening was significantly stronger in the rocuronium 0.9 mg/kg IBW group than in the 0.3 mg/kg IBW group. Figure 3 depicts the dose–response dynamic of each patient.

Secondly, we investigated if the rocuronium dosage is associated with differences in drug effect over time. There was no significant ANOVA difference (*p* = 0.937) between the LMM with and without group interaction, while the addition of group interaction in the vocal cord models significantly increased the prediction of the ‘ST response amplitude’ (*p* < 0.001).

**Fig. 4:**
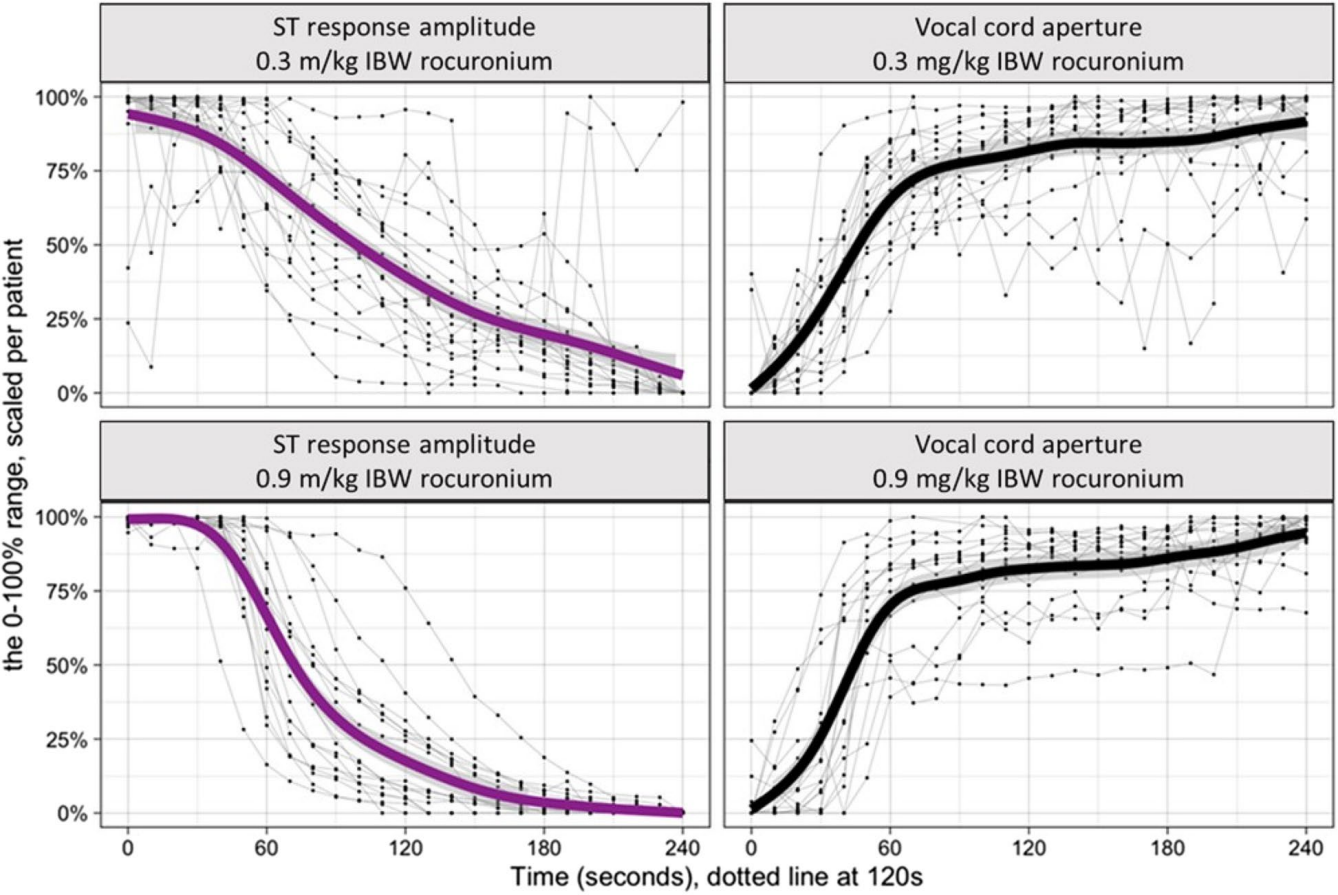
Dose–response dynamic per group. The figure presents the dose–response dynamics for both groups: rocuronium: 0.3 mg/kg IBW (upper plots) and 0.9 mg/kg IBW (lower plots). ‘ST response amplitude’ and ‘vocal cord aperture’ are scaled from minimum 0 to maximum 100% for each patient and are shown on the left (magenta) and right (black) vertical axes, respectively. The time elapsed in seconds since the administration of rocuronium is plotted along the horizontal axis. Individual patient responses are represented by thin gray lines, revealing the inter-patient variability. The solid thick magenta and black lines indicate the central trend of the group’s response over time, modeled using nonlinear Generalized Additive Models. Firstly, we comment about the central cohort’s trend under both dosages: in the ‘ST response amplitude’ plots, the 0.9 mg/kg IBW dose group shows a steeper initial decline, compared to the 0.3 mg/kg IBW group. On the other side, the time-dependent vocal cord opening was not significantly different across the two dosages. Secondly, regarding inter-patient variability, indicated by the spread of the gray lines around the thick lines, both groups exhibit less variability using 0.9 mg/kg IBW rocuronium compared to the 0.3 mg/kg IBW group

Consequently, from these results and the visual examination of Fig. 4, we can derive that the ‘ST response amplitude’ decreased steeper in patients after 0.9 mg/kg IBW compared to 0.3 mg/kg IBW, while we observed no significant differences for ‘vocal cord aperture’ over time. Furthermore, the inter-patient variability in both, ‘response amplitude’ and ‘vocal cord aperture’, decreased in the 0.9 mg/kg IBW rocuronium group.

## Discussion

We investigated whether the electromyographic ST is a suitable stimulation pattern to predict vocal cord opening for tracheal intubation. We found that there is a strong negative correlation between decreasing ST response amplitude and increasing vocal cord aperture. The correlation was stronger after 0.9 mg/kg IBW rocuronium compared to 0.3 mg/kg IBW (primary endpoint). In addition, we found that the response amplitude’s decrease was steeper after 0.9 compared to 0.3 mg/kg IBW rocuronium, whereas we saw no significant dose-dependent effect on the vocal cord aperture over time, although the inter-patient variability was lower in the high-dose rocuronium group (secondary endpoint).

The European Society of Anaesthesiology and Intensive Care recommends the use of NMBDs to facilitate tracheal intubation and to reduce the risk for pharyngeal and laryngeal injuries [1]. However, the ideal point in time for tracheal intubation after NMBD administration is difficult to determine. A reason for this might be that the established stimulation patterns such as TOF or PTC are limited or just not suited to best predict vocal cord aperture, e.g. because the stimulation pattern’s timing or its significance is not appropriate. The benefits of monitoring over the anaesthesiologist’s subjective assessment of ‘readiness for intubation’ may then appear only marginal in clinical practice. It indeed is difficult to describe a ‘static’ process such as the opening of the vocal cords (a non-evoked response of muscle paralysis resulting from rocuronium) with a ‘dynamic’ parameter (evoked response; neuromuscular function due to electrical stimulation) such as the TOF stimulation pattern, simply due to physiological and methodological reasons [12]. Thus, the calculation of a ratio represents a relative value only, not a measured ‘absolute’ value; however, the latter is what is needed to describe the static vocal cord opening process. Consequently, the Pearson analysis in this study showed consistency in the decreasing of the ST simultaneously with the increasing of vocal-fold area, while Fig. 4 demonstrated the underlying non-linearities in the data. However, the ST response amplitude was a reliable predictor. It demonstrated a quantifiable, measured value every 10 s, which seems an adequate time interval for tracheal intubation.

The predictive power of the electromyographic ST response amplitude was significantly stronger in patients who had received 0.9 mg/kg IBW rocuronium than in those who received 0.3 mg/kg IBW. The effective dose 95 (ED95), which describes a 95% neuromuscular block, is 0.3 mg/kg for rocuronium. The recommended dosage for normal tracheal intubation usually corresponds to 2 times ED95, which is 0.6 mg/kg. Higher dosages like 0.9 mg/kg (3 times ED95) or more are used for a rapid sequence induction to guarantee a short time of onset with ‘good intubation conditions’ [13]. Thus, in this study we compared a known “low” dose (0.3 mg/kg IBW) with a known “high” dose (0.9 mg/kg IBW) of rocuronium for standard tracheal intubation (the dose of 0.6 mg/kg is recommended for routine tracheal intubation). Therefore, the stronger correlation and the lower inter-patient variability in the high-dose group were expected; however, we also focused on the impact of the NMBD’s dosage on the vocal cord opening as a static parameter, which showed comparable results between the groups. This was very interesting, as it demonstrates that ED95 is sufficient for complete vocal cord aperture, while reflexes (e.g. coughing) are probably maintained and a quick recovery of the diaphragm and the other muscles can be expected. Although this is likely not important for most patients, there might be situations where these thoughts are of note (short duration of surgery, difficult airway management). An explanation might be that laryngeal muscles, which are responsible for vocal cord aperture, are very sensitive to NMBDs [14]. Similar observations regarding the vocal cord aperture were made by others, who stated that the vocal cords’ position and the degree of movement were adequate for tracheal intubation after 0.3 mg/kg rocuronium, although complete muscle relaxation, defined as TOF count ≤ 1 was not reached in 17–34% of the patients [13, 15].

This study did not assess the overall (and subjective) intubation conditions but focused on the relationship between response amplitude and vocal cord aperture only. The latter is an important requirement for safe and injury-free tracheal intubation; however, it certainly does not cover all aspects of ‘ideal intubation conditions’, as the relaxation of the masseter, the pharyngeal muscles, or the diaphragm were not investigated. Although this could be considered a limitation, it should be noted that ‘optimal intubation conditions’ are methodologically very difficult to evaluate, as there is no standardized assessment. Different classifications and scores such as Cormack and Lehane, the Fuchs-Buder score, or the intubation difficulty scale, descriptions of the vocal cords’ positioning, or the activity of the diaphragm were normally used to judge the intubation conditions, however, this is always a subjective assessment, whereas we objectively quantified both the vocal cord aperture, and the response amplitude [16–18].

Another aspect is that ‘intubation conditions’ are influenced by the depth of anaesthesia and the used substances. Thus, remifentanil and propofol, which both were administered for induction of anaesthesia and during the four minutes of vocal cord video documentation after rocuronium was given, may have had an additive effect on the vocal cord aperture. Data have shown that the airway management can be simplified by using opioids, however, this effect is less pronounced in combination with high NMBD doses [19]. Thus the effect of remifentanil on vocal cord aperture may not have been the same in both groups. Apart from the fact that we cannot dispense the induction dose due to ethical reasons, we mitigate its effect by standardization of the effective remifentanil and propofol doses, as well as by monitoring the depth of anaesthesia via Narcotrend Index.

The comparatively small sample size in our study could also be interpret as limitation. However, the strong correlations and the *p*-value < 0.00001 let us assume that rocuronium and the ST amplitude indeed correlate. In addition, the role of our bootstrapping methodology was double: 1) it respects the statistical assumptions that the samples should be clustered-independent, i.e. in every iteration only one sample per patient is analysed; and 2) it produces a distribution of correlation coefficients, as such it is from the statistical point of view more reliable than the usual single coefficient estimation.

Lastly, all investigated patients were either of normal weight or only slightly obese. It is of note that the response amplitude baseline value is usually decreased in (very) obese patients due to a higher tissue impedance. Although we did not focus on this, it is possible that the predictive power of the ST response amplitude might be reduced in these patients.

In summary, our results demonstrated that the quantitative, electromyographic ST response amplitude is suitable to predict vocal cord aperture in real-time after rocuronium administration. A higher rocuronium dose caused a stronger correlation, a lower inter-patient variability, and a steeper ST decrease, however, the dosage had no effect on the maximum vocal cord aperture in the investigated four minutes time interval. Regarding the ease of use and interpretation of data, we hope that these objective measurement techniques will be used in further studies.

## Data Availability

Data supporting this study are included within the article. Please contact the corresponding author for further questions.

## Abbreviations

ANOVA: Analysis of Variance
ASA: American Society of Anesthesiologists
ED95: Effective dose 95
IBW: Ideal body weight
IQR: Interquartile range
LMM: Linear multilevel models
M: Musculus
MAX: Maximum
MIN: Minimum
NMBDs: Neuromuscular blocking drugs
PS: Physical status
PTC: Post-tetanic count
ST: Single twitch
SD: Standard deviation
T: Twitch
TOF: Train-of-four
